# A multicentre retrospective observational study on Polish experience of pirfenidone therapy in patients with idiopathic pulmonary fibrosis: the PolExPIR study

**DOI:** 10.1186/s12890-020-1162-6

**Published:** 2020-05-04

**Authors:** Sebastian Majewski, Adam J. Białas, Małgorzata Buchczyk, Paweł Gomółka, Katarzyna Górska, Hanna Jagielska-Len, Agnieszka Jarzemska, Ewa Jassem, Dariusz Jastrzębski, Aleksander Kania, Marek Koprowski, Rafał Krenke, Jan Kuś, Katarzyna Lewandowska, Magdalena M. Martusewicz-Boros, Kazimierz Roszkowski-Śliż, Alicja Siemińska, Krzysztof Sładek, Małgorzata Sobiecka, Karolina Szewczyk, Małgorzata Tomczak, Witold Tomkowski, Elżbieta Wiatr, Dariusz Ziora, Beata Żołnowska, Wojciech J. Piotrowski

**Affiliations:** 1 Department of Pneumology and Allergy, Medical University of Lodz, Lodz, Poland; 2 Department of Pathobiology of Respiratory Diseases, Medical University of Lodz, Lodz, Poland; 3 Department of Lung Diseases and Tuberculosis, School of Medicine with the Division of Dentistry in Zabrze, Medical University of Silesia, Katowice, Poland; 4 Department of Pulmonology, Jagiellonian University Medical College, Cracow, Poland; 5 Department of Internal Medicine, Pulmonary Diseases and Allergy, Medical University of Warsaw, Warsaw, Poland; 6 Clinical Department of Lung Diseases, K. Marcinkowski University Hospital, Zielona Gora, Poland; 7 Department of Pneumonology, Oncology and Tuberculosis, Kuyavian and Pomeranian Pulmonology Centre, Bydgoszcz, Poland; 8 Department of Allergology and Pneumonology, Medical University of Gdansk, Gdansk, Poland; 9 Department of Civilization Diseases and Lung Diseases, John Paul II Specialist Hospital, Cracow, Poland; 10 1st Department of Lung Diseases, National Tuberculosis and Lung Diseases Research Institute, Warsaw, Poland; 11 3rd Lung Diseases and Oncology Department, National Tuberculosis and Lung Diseases Research Institute, Warsaw, Poland; 12 Department of Pulmonology, E.J. Zeyland Wielkopolska Center of Pulmonology and Thoracic Surgery, Poznan, Poland

**Keywords:** Pirfenidone, Idiopathic pulmonary fibrosis, Real-world data, Efficacy, Safety, Poland

## Abstract

**Background:**

Pirfenidone is an antifibrotic agent approved for the treatment of idiopathic pulmonary fibrosis (IPF). The drug is available for Polish patients with IPF since 2017. The PolExPIR study aimed to describe the real-world data (RWD) on the Polish experience of pirfenidone therapy in IPF with respect to safety and efficacy profiles.

**Methods:**

This was a multicentre, retrospective, observational study collecting clinical data of patients with IPF receiving pirfenidone from January 2017 to September 2019 across 10 specialized pulmonary centres in Poland. Data collection included baseline characteristics, pulmonary function tests (PFTs) results and six-minute walk test (6MWT). Longitudinal data on PFTs, 6MWT, adverse drug reactions (ADRs), treatment persistence, and survival were also collected up to 24 months post-inclusion.

**Results:**

A total of 307 patients receiving pirfenidone were identified for analysis. The mean age was 68.83 (8.13) years and 77% were males. The median time from the first symptoms to IPF diagnosis was 15.5 (9.75–30) months and from diagnosis to start of pirfenidone treatment was 6 (2–23) months. Patients were followed on treatment for a median of 17 (12–22.75) months. Seventy-four patients (24.1%) required dose adjustments and 35 (11.4%) were chronically treated with different than the full recommended dose. A total of 141 patients (45.92%) discontinued therapy due to different reasons including ADRs (16.61%), death (8.79%), disease progression (6.51%), patient’s own request (5.54%), neoplastic disease (3.91%) and lung transplantation (0.33%). Over up to 24 months of follow-up, the pulmonary function remained largely stable. The median annual decline in forced vital capacity (FVC) during the first year of pirfenidone therapy was −20 ml (−200–100) and during the second year was −120 ml (−340–30). Over a study period, 33 patients (10.75%) died.

**Conclusions:**

The PolExPIR study is a source of longitudinal RWD on pirfenidone therapy in the Polish cohort of patients with IPF supporting its long-term acceptable safety and efficacy profiles and reinforce findings from the previous randomised clinical trials and observational studies.

## Background

Idiopathic pulmonary fibrosis (IPF) is a chronic, progressive, fibrotic lung disease, one of the most common among a heterogeneous group of interstitial lung diseases (ILDs). The natural history of the disease is characterized by inevitable, progressive decline of lung function, reduction in exercise tolerance, quality of life and premature death [1]. IPF is known to have outcomes worse than many cancers, with a median survival of 3 to 5 years after diagnosis, though the disease course varies significantly in individuals [2, 3]. Two molecules, pirfenidone and nintedanib, have been shown to slow the disease progression limiting the decline of lung function in patients with IPF [4–6]. Both antifibrotics are recognized as an actual standard of pharmacological treatment of the disease [7]. Despite early drug registrations in Europe (pirfenidone in 2011 and nintedanib in 2015), lack of reimbursement for antifibrotics in Poland led to significant limitations in wide access to antifibrotic therapy for Polish patients with IPF [8]. Recent changes in the reimbursement policy for antifibrotics in Poland have resulted in much wider availability of pirfenidone (since January 2017) and nintedanib (since March 2018) for the treatment of Polish patients with IPF.

Randomised clinical trials (RCTs) on pirfenidone therapy in IPF provided important information on its efficacy and safety profiles. These trials proved that pirfenidone treatment in IPF possesses acceptable safety, significantly reduces the rate of decline of forced vital capacity (FVC) and its use is associated with decreased mortality [9]. However, the follow-up period of pirfenidone RCTs lasted only 52–72 weeks [4, 5, 10]. Moreover, strict inclusion and exclusion criteria of RCTs may limit the possibility of generalization of the results to the real-world setting clinical practice. Therefore, real-world data (RWD) studies based on data obtained outside the context of RCTs generated during routine clinical practice provide additional pieces of evidence on the long-term efficacy and safety of particular intervention in the broader real-world patients populations.

Up to date, several RWD studies were published supplementing previous evidence of RCTs on the efficacy and safety of pirfenidone in the treatment of heterogenous IPF patients populations, however the scientific significance of many of those studies suffer from a small number of patients included for analysis (<100 patients) [11–30]. Moreover, no data exist describing the clinical efficacy and safety of long-term pirfenidone use in patients with IPF in Poland.

The PolExPIR was a multicentre, retrospective, observational study collecting clinical data of patients with IPF receiving pirfenidone from January 2017 to September 2019 across 10 specialized pulmonary centres in Poland. The aim of the project was to provide for the first time longitudinal data on clinical outcomes of pirfenidone therapy in Polish cohort of patients with IPF under real-world conditions.

## Methods

### Study cohort

In this study, a targeted population of Polish patients with IPF receiving pirfenidone treatment in the setting of therapeutic program refunded by the National Health Fund (NHF) for patients with the mild-to-moderate disease was analysed. Main inclusion criteria for the NHF therapeutic program were: a multidisciplinary team (MDT) confident diagnosis of IPF according to 2011 international diagnostic guidelines [2], FVC above 50% of predicted value and transfer factor of the lung for carbon monoxide (T_LCO_) above 30% of predicted value. All patients eligible for NHF therapeutic program were required a regular follow-up with safety and efficacy assessments. A mandatory re-evaluation of PFTs every 6 months since drug initiation was demanded and disease progression defined as a significant FVC decline (≥10% of predicted value) over the first 12 months or over consecutive (after 12-months treatment) 6 months intervals has been established as an exclusion criterion from continuation of pirfenidone therapy (drug stop rule). The PolExPIR study enrolled all patients with IPF receiving pirfenidone therapy for at least 12 months in the setting of the NHF therapeutic program and those discontinuing pirfenidone for any reason regardless of timing. The study inclusion period lasted from January 2017 to September 2019. The local ethics committee needed no approval, as the study was retrospective, patients’ data were anonymized and pirfenidone was already approved in Poland.

### Data collection

The PolExPIR study collected clinical data of patients with IPF receiving pirfenidone across 10 specialized pulmonary centres in Poland responsible for the diagnosis and management of a broad spectrum of ILDs patients and the supervision of antifibrotic therapy of patients with IPF in the frame of NHF therapeutic program. Retrospective clinical data collection included baseline characteristics, data on diagnosis, previous treatment for IPF, supplemental oxygen use, pulmonary function tests (PFTs) results including spirometry and T_LCO_ measurements, and six-minute walk test (6MWT). Longitudinal data on PFTs, 6MWT, adverse drug reactions (ADRs), treatment persistence, and survival were also collected up to 24 months post-inclusion. To avoid the possible bias in PFTs data interpretation associated with the use of various reference equations and various reference values, the measurements performed in different centres were reported as absolute values and then expressed as the percentages of predicted using the Global Lung Function Initiative (GLI) reference values [31, 32].

### Statistical analysis

Data were analysed using the R software for MacOS (R Core Team, 2019, Vienna, Austria). Normality of data distribution was tested with the Shapiro-Wilk test. Continuous data are expressed as mean with standard deviation (SD) for normally distributed data or as median with interquartile range (IQR) for non-normally distributed data. Categorical data are presented as absolute numbers and relative frequencies. For the longitudinal analysis, all available data were included. Data were analysed using the Wilcoxon signed-rank test. For the longitudinal efficacy assessment of all the functional parameters (FVC, T_LCO_ and 6MWT) the difference between the initial and the last data available for the analysis was used. The dynamics of pulmonary function was analysed as a difference between the timepoints specified for each comparison separately and described in the results section. Additional subanalysis of the functional parameters changes in the 6-months intervals was also performed. For the purpose of subanalysis of efficacy data patients were divided based on the rate of changes in FVC % of predicted (∆FVC) in 6-months intervals into the following groups: significant improvement (∆FVC >10%), marginal improvement (10% ≥ ∆FVC>5%), stabilization (+5% ≥ ∆FVC>-5%), marginal decline (−5% ≥ ∆FVC>-10%), and significant decline (∆FVC ≤ -10%). In terms of the rate of changes in T_LCO_ % of predicted (∆T_LCO_) in 6-months intervals, patients were divided into the following groups: significant improvement (∆T_LCO_ >15%), stabilization (+15% ≥ ∆T_LCO_>-15%), and significant decline (∆T_LCO_ ≤ -15%). The graphs were performed using GraphPad Prism 8 (GraphPad Software, La Jolla, San Diego, CA, USA).

## Results

### Baseline characteristics

From January 2017 to September 2019 a total of 307 patients receiving pirfenidone in the setting of the NHF therapeutic program across 10 participating sites were identified for analysis. Patients were followed on pirfenidone treatment for a median of 17 months (12–22.75). Summary of baseline characteristics of study participants is shown in Table 1. The mean age at the onset of pirfenidone treatment was 68.83 (8.13) years, and 77% of the patients were males. The majority of patients were former smokers (68.73%) however, more than 4% of IPF patients (4.23%) were still actively smoking at the time of enrolment for antifibrotic therapy. In almost 85% of subjects IPF was diagnosed based on the presence of a definite usual interstitial pneumonia (UIP) pattern on the high-resolution computed tomography (HRCT) and the remaining of patients required a combination of HRCT and lung biopsy for the definite IPF diagnosis, see Table 1. Seventeen patients with definite UIP pattern on HRCT had undergone a surgical lung biopsy (SLB) prior to the publication of 2011 diagnostic guidelines for IPF [2]. Transbronchial lung biopsy (TBLB) was used in the diagnostic work-up in as many as 9 patients but the results were diagnostic for UIP only in 2 cases. Transbronchial lung cryobiopsy (TBLC) was used for confirmation of IPF diagnosis only in 2 patients. The median latency period from the first symptoms to IPF diagnosis was 15.5 months (9.75–30) and from diagnosis to start of pirfenidone treatment was 6 months (2-23) . At baseline, the median FVC was 77.08% of predicted (67.02–88.43) and the median T_LCO_ was 52.24% of predicted (42.56–64.55). The mean partial arterial oxygen pressure (PaO_2_) was 69 mmHg (*n* = 207). The median baseline distance covered in the 6MWT was 490 m (400–540) (*n* = 165) with a median of 7% desaturation (4-12) during the test. The majority of patients (95.76%) belonged to stage I and II according to the multidimensional prognostic staging system for IPF (Gender, Age, and Physiology - GAP index) [33]. Fifty-one patients (16.61%) were using home oxygen therapy and 21 patients (6.84%) were using portable sources of oxygen at the onset of pirfenidone treatment. The majority of patients (78.18%) had not been treated for IPF before pirfenidone initiation, whereas approximately 10% of subjects had been treated in the past with corticosteroids (CS) or participated in various clinical trials.

**Table 1:** Characteristics of study participants

**Number of patients,** n (%)	307 (100)
City of Bydgoszcz, n (%)	15 (4.89)
City of Cracow (2 centres), n (%)	54 (17.59)
City of Gdansk, n (%)	26 (8.47)
City of Lodz, n (%)	40 (13.03)
City of Poznan, n (%)	9 (2.93)
City of Warsaw (2 centres), n (%)	75 (24.43)
City of Zabrze, n (%)	75 (24.43)
City of Zielona Gora, n (%)	13 (4.23)
Sex, male/female, n (%)	237 (77.2)/70 (22.8)
Age (years), mean (SD)	68.83 (8.13)
**Smoking history**	
Never smokers, n (%)	72 (23.45)
Former smokers, n (%)	211 (68.73)
Active smokers, n (%)	13 (4.23)
No data, n (%)	11 (3.58)
Pack-years, median (IQR)	30 (15–40)
**Comorbidities**	
Hypertension, n (%)	192 (62.54)
Coronary artery disease, n (%)	98 (31.92)
Hyperlipidaemia, n (%)	126 (41.04)
Atrial fibrillation, n (%)	39 (12.70)
Heart failure, n (%)	53 (17.26)
Gastroesophageal reflux disease, n (%)	114 (37.13)
Diabetes, n (%)	77 (25.08)
Emphysema, n (%)	51 (16.61)
Depression, n (%)	28 (9.12)
Obstructive sleep apnoea, n (%)	23 (7.49)
Benign prostate hypertrophy, n (%)	84 (27.36)
Neoplastic disease history, n (%)	24 (7.82)
Hypothyroidism, n (%)	27 (8.79)
Osteoarthritis of the spine, n (%)	24 (7.82)
**Diagnosis of IPF**	
Radiologic UIP pattern, n (%)	260 (84.69)
Radiologic possible UIP pattern + SLB, n (%)	23 (7.49)
Radiologic possible UIP pattern + TBLC, n (%)	2 (0.65)
Radiologic inconsistent for UIP pattern + SLB, n (%)	3 (0.98)
Radiologic UIP pattern + SLB, n (%)	17 (5.54)
TBLB, n (%)	9 (2.93)
TBLB result diagnostic for UIP, n (%)	2/9 (22.22)
**Time from first symptoms to diagnosis** (months), median (IQR)	15.5 (9.75–30)
**Time from diagnosis to start of pirfenidone therapy** (months), median (IQR)	6 (2–23)
**Pulmonary function**	
FEV_1_ (l), median (IQR)	2.29 (1.92–2.69)
FEV_1_ (% of predicted), median (IQR)	87.28 (71.88–90.42)
FVC (l), median (IQR)	2.88 (2.35–3.39)
FVC (% of predicted), median (IQR)	77.08 (67.02–88.43)
T_LCO_ (mmol/min/kPa), median (IQR)	4.02 (3.2–5.03)
T_LCO_ (% of predicted), median (IQR)	52.24 (42.56–64.55)
**Blood oxygenation**	
SpO_2_ at rest (%), median (IQR)	95 (93–96)
PaO_2_ at rest (n = 207), (mmHg), mean (SD)	69.06 (9.75)
**6MWT**	
Distance (n = 165), (meters), median (IQR)	490 (400–540)
Desaturation, (∆%), median (IQR)	7 (4–12)
**GAP score**, median (IQR)	3 (3–4)
**GAP index:**	
Stage I, n (%)	170 (55.37)
Stage II, n (%)	124 (40.39)
Stage III, n (%)	13 (4.23)
**Oxygen therapy**	
Home oxygen therapy, n (%)	51 (16.61)
Ambulatory oxygen therapy (portable sources), n (%)	21 (6.84)
**IPF treatment in the past before initiation of pirfenidone**	
No treatment, n (%)	240 (78.18)
CS, n (%)	33 (10.75)
NAC, n (%)	1 (0.33)
CS + NAC, n (%)	0 (0)
CS + NAC + AZA, n (%)	1 (0.33)
Clinical trial, n (%)	32 (10.42)

*Abbreviations*: *UIP* usual interstitial pneumonia, *SLB* surgical lung biopsy, *TBLC* transbronchial lung cryobiopsy, *TBLB* transbronchial lung biopsy, *FEV*_*1*_ forced expiratory volume in 1 s, *FVC* forced vital capacity, *IPF* idiopathic pulmonary fibrosis, *T*_*LCO*_ transfer factor of the lung for carbon monoxide, *SpO*_*2*_ percutaneous oxygen saturation, *PaO*_*2*_ partial pressure of arterial oxygen, *6MWT* six-minute walk test, *GAP* gender, age, and 2 physiology variables (FVC and T_LCO_), *CS* corticosteroids, *NAC* N–acetylcysteine, *AZA* azathioprine

### Tolerability, safety and drug persistence assessment

Table 2 summarizes the tolerability and ADRs of pirfenidone treatment. Seventy-four patients (24.1%) required dose adjustments including intermittent drug interruption and/or reduction of dosing to continue adherence to treatment and 35 (11.4%) were chronically treated with a different than the full recommended dose. The most frequent ADRs were fatigue (35.83%), decreased appetite (34.2%), weight loss (32.57%), cough (28.66%), nausea (24.43%), dyspepsia (23.13%), skin rash (18.89%) and photosensitivity reactions (17.59%).

**Table 2:** Tolerability and adverse drug reactions (ADRs) of pirfenidone therapy

**Full dose treatment, n (%)**	272 (88.6)
**Different than full dose treatment, n (%)**	35 (11.4)
**Intermittent drug interruption and/or reduction of dosing, n (%)**	74 (24.1)
**ADRs**	
Nausea, n (%)	75 (24.43)
Decreased appetite, n (%)	105 (34.2)
Diarrhoea, n (%)	33 (10.75)
Vomiting, n (%)	15 (4.89)
Dyspepsia, n (%)	71 (23.13)
Loss of weight, n (%)	100 (32.57)
Cough, n (%)	88 (28.66)
Fatigue, n (%)	110 (35.83)
Dizziness, n (%)	42 (13.68)
Skin rash, n (%)	58 (18.89)
Photosensitivity, n (%)	54 (17.59)
Elevated liver enzymes, n (%)	9 (2.93)
Sleep disturbances, n (%)	49 (15.96)
Other, n (%)	36 (11.73)

*Abbreviations*: *ADRs* adverse drug reactions

**Fig. 1:**
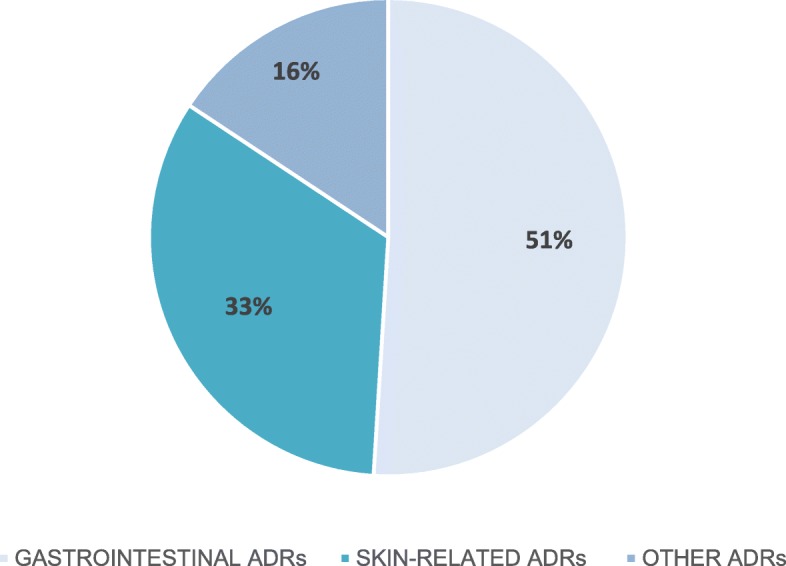
Adverse drug reactions (ADRs) categories leading to pirfenidone treatment discontinuation

Over the median time of 17 months (12–22.75) of pirfenidone exposition, a total of 141 patients (45.9%) discontinued therapy due to different reasons including ADRs (16.6%), death (8.7%), disease progression (6.5%), patient’s own request (5.5%), diagnosis of neoplastic disease (3.9%) and lung transplantation (0.3%), see Table 3. Among ADRs leading to treatment discontinuation the most common were gastrointestinal ADRs (51%) and skin-related ADRs (33%), see Fig. 1. Over a study period, 33 patients (10.75%) died. IPF-related death occurred in 21 patients (63.64%), cardiovascular-related death in 2 patients (6.06%), neoplastic disease-related death in 3 patients (9.09%) and unknown or other cause of death in 7 patients (21.21%).

**Table 3:** Treatment persistence

**Pirfenidone exposition,** (months)**,** median (IQR)	17 (12–22.75)
**Reasons for treatment discontinuation**	
ADRs, n (%)	51 (16.61)
Disease progression, n (%)	20 (6.51)
Death, n (%)	27 (8.79)
Patient’s decision, n (%)	17 (5.54)
Lung transplantation, n (%)	1 (0.33)
Neoplastic disease, n (%)	12 (3.91)
Other, n (%)	13 (4.23)

*Abbreviations*: *ADRs* adverse drug reactions

### Longitudinal efficacy assessment

**Fig. 2:**
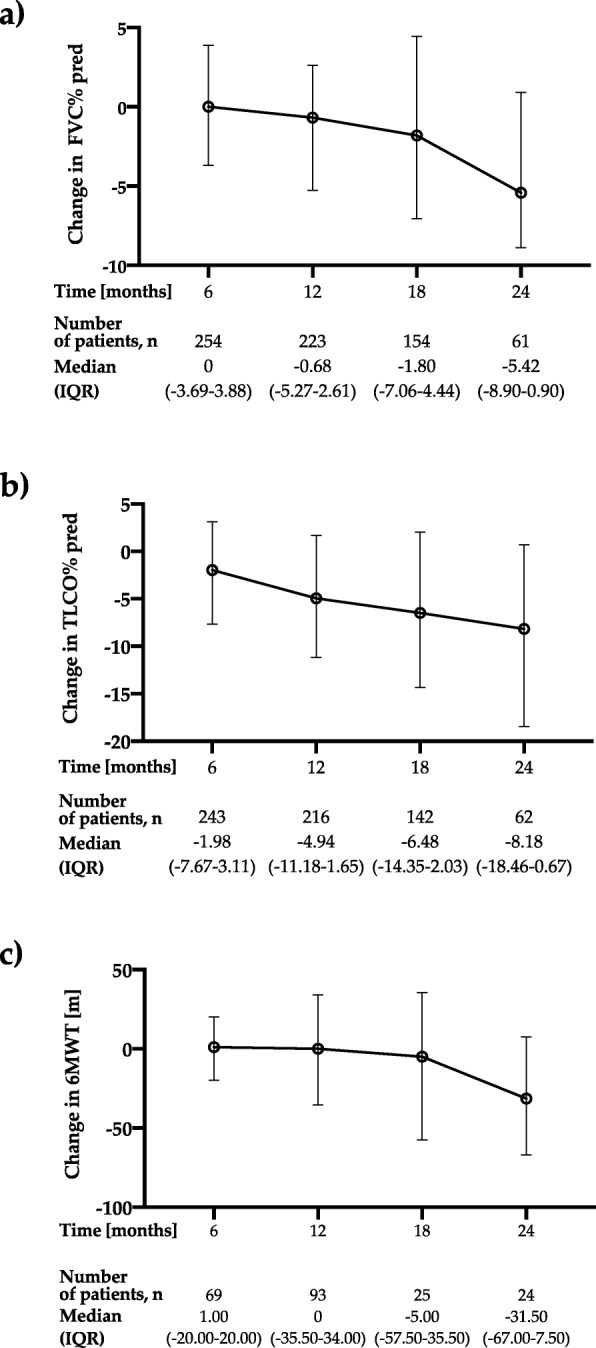
Change over 24 months of follow-up in: **a)** FVC % of predicted; **b)** T_LCO_ % of predicted; **c**) 6MWT distance. Change from baseline was calculated as a follow-up time point value minus the baseline value, therefore negative value indicates a decrease from baseline. Data are presented as median (IQR) values. Abbreviations: FVC – forced vital capacity, T_LCO_ – transfer factor of the lung for carbon monoxide, 6MWT – six-minute walk test

The median annual decline in forced vital capacity (FVC) during the first year of pirfenidone treatment was −20 (−200–100) ml (*n* = 226) and during the second year was −120 (−340–30) ml (*n* = 61). The median change from baseline in % of predicted FVC was −0.68% (−5.27–2.61) at month 12 and −5.42% (−8.90–0.90) at month 24, see Fig. 2a.

Meanwhile, the median T_LCO_ decline was −0.34 (−0.83–0.14) mmol/min/kPa in the first year of pirfenidone treatment (*n* = 222) and −0.57 (−1.11–0.25) mmol/min/kPa in the second year of therapy (*n* = 61). The median change from baseline in % of predicted T_LCO_ was −4.94% (−11.18–1.65) at month 12 and −8.18% (−18.46–0.67) at month 24, see Fig. 2b. The median change from baseline in 6MWT distance during pirfenidone treatment was 0.00 (−35.5–34.00) meters (*n* = 93) at month 12 and −31.5 (−67.00–7.5) meters (*n* = 24) at month 24, see Fig. 2c.

**Fig. 3:**
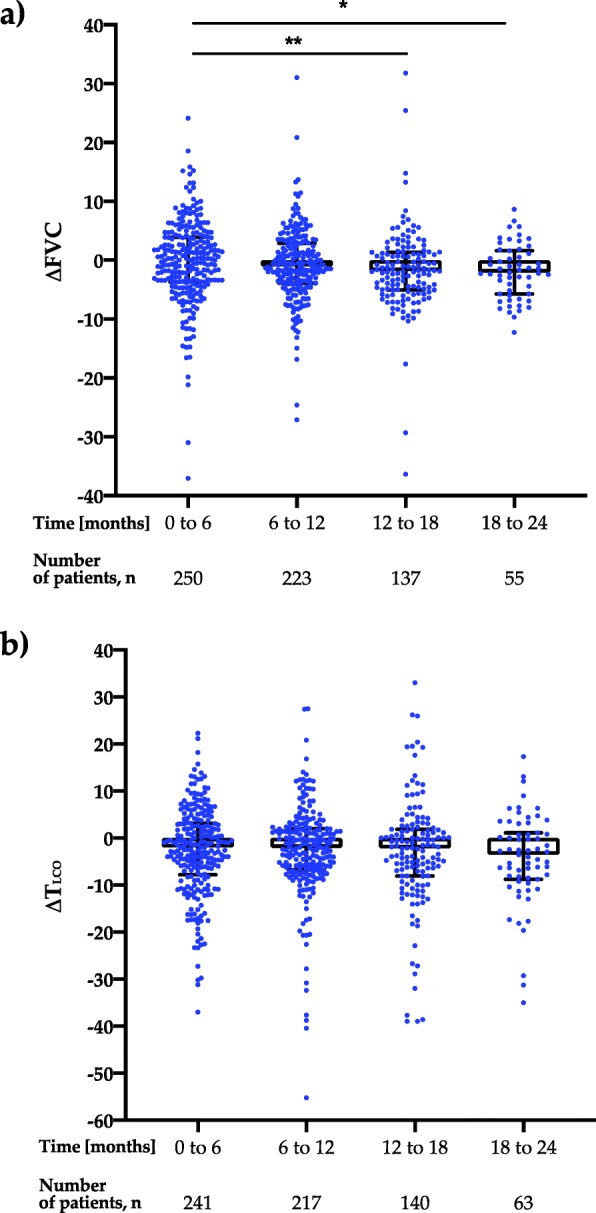
Longitudinal 6-months interval change during pirfenidone treatment in **a**) FVC % of predicted (ΔFVC); **b**) T_LCO_ % of predicted (ΔT_LCO_). Data are presented as median (IQR) values. Notes: *p < 0.05, **p < 0.01. Abbreviations: FVC – forced vital capacity, T_LCO_ – transfer factor of the lung for carbon monoxide

The longitudinal analysis of changes in FVC % of predicted and T_LCO_ % of predicted in 6-months intervals is shown in Fig. 3 and Table 4. The significant differences were noted in the rate of decline of FVC % of predicted in the second year of pirfenidone therapy, namely between 12 and 18 months interval as well as between 18 and 24 months interval compared to the first 0–6 months interval (*p* < 0.05). No significant difference was noted in the rate of decline of T_LCO_ % of predicted in the interval analysis during 24 months of pirfenidone treatment.

**Table 4:** Longitudinal 6–months interval change during pirfenidone treatment in FVC % of predicted (ΔFVC) and T_LCO_ % of predicted (ΔT_LCO_). Data are presented as median (IQR) values

	0–6 months	6–12 months	12–18 months	18–24 months	p _**(6–12 vs 0–6)**_	p _**(12–18 vs 0–6)**_	p _**(18–24 vs 0–6)**_
**∆FVC**	0 (−3.69–3.93)	−1.06 (−3.90–2.90)	−1.84 (−5.04–1.37)	−2.10 (−5.73–1.63)	0.13	< 0.01	< 0.05
**∆T**_**LCO**_	−2.02 (−7.81–3.09)	−2.11 (−6.79–1.96)	−2.17 (−8.07–1,84)	−3.56 (−8.77–1.12)	0.98	0.24	0.22

*Abbreviations*: *FVC* forced vital capacity, *T*_*LCO*_ transfer factor of the lung for carbon monoxide

The 6-months interval subanalysis of FVC and T_LCO_ data is shown in Table 5.

**Table 5:** Patient distribution in relation to longitudinal 6–months interval change in FVC % of predicted (ΔFVC) and T_LCO_ % of predicted (ΔT_LCO_)

ΔFVC	0–6 months	6–12 months	12–18 months	18–24 months
significant improvement (∆FVC >10%) marginal improvement (10% ≥ ∆FVC>5%) stabilization (+5% ≥ ∆FVC>-5%) marginal decline (−5% ≥ ∆FVC>-10%) significant decline (∆FVC ≤ -10%) n	14 (5.6%) 35 (14%) 152 (60.8%) 31 (12.4%) 18 (7.2%) 250	7 (3.1%) 25 (11.2%) 152 (68.2%) 28 (12.6%) 11 (4.9%) 223	4 (2.9%) 9 (6.6%) 89 (65%) 31 (22.6%) 4 (2.9%) 137	0 (0%) 2 (3.6%) 36 (65.5%) 16 (29.1%) 1 (1.8%) 55
∆T_LCO_	0–6 months	6–12 months	12–18 months	18–24 months
significant improvement (∆T_LCO_ >15%) stabilization (+15% ≥ ∆T_LCO_>-15%) significant decline (∆T_LCO_ ≤ -15%) n	4 (1.7%) 210 (87.1%) 27 (11.2%) 241	4 (1.8%) 197 (90.8%) 16 (7.4%) 217	8 (5.7%) 119 (85%) 13 (9.3%) 140	1 (1.6%) 55 (87.3%) 7 (11.1%) 63

*Abbreviations*: *FVC* forced vital capacity, *T*_*LCO*_ transfer factor of the lung for carbon monoxide

Longitudinal change in FVC and T_LCO_ varied among the individual cases in our study. Change in FVC in the majority of patients (range of 61–68% depending on the interval analysed) was stable, and only in the minority of them (range 4–14% and 0–6%) showed marginal or significant improvement, respectively. Marginal or significant decline was observed likewise only in the minority of patients (range 12–29% and 2–7%, respectively). In terms of change of T_LCO_ values longitudinal evaluation confirmed stabilization in the majority of patients (range 85–91% depending on the interval analysed) and significant improvement or significant decline only in the minority of them (range 2–6% and 7–11%, respectively).

**Fig. 4:**
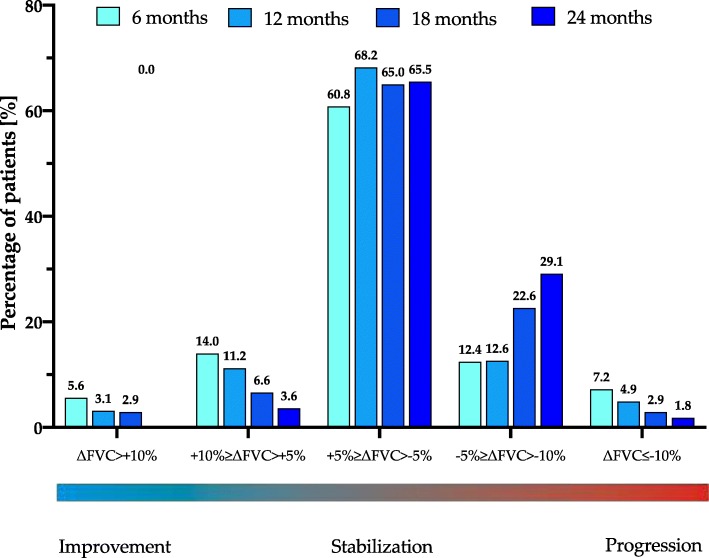
Percentage of patients with IPF experiencing significant (∆FVC >10%) or marginal (10% ≥ ∆FVC>5%) improvement, stabilization (+5% ≥ ∆FVC>-5%), and marginal (−5% ≥ ∆FVC>-10%) or significant (∆FVC ≤ -10%) decline based on the rate of changes of FVC % of predicted (∆FVC) in the 6-months intervals during pirfenidone treatment

**Fig. 5:**
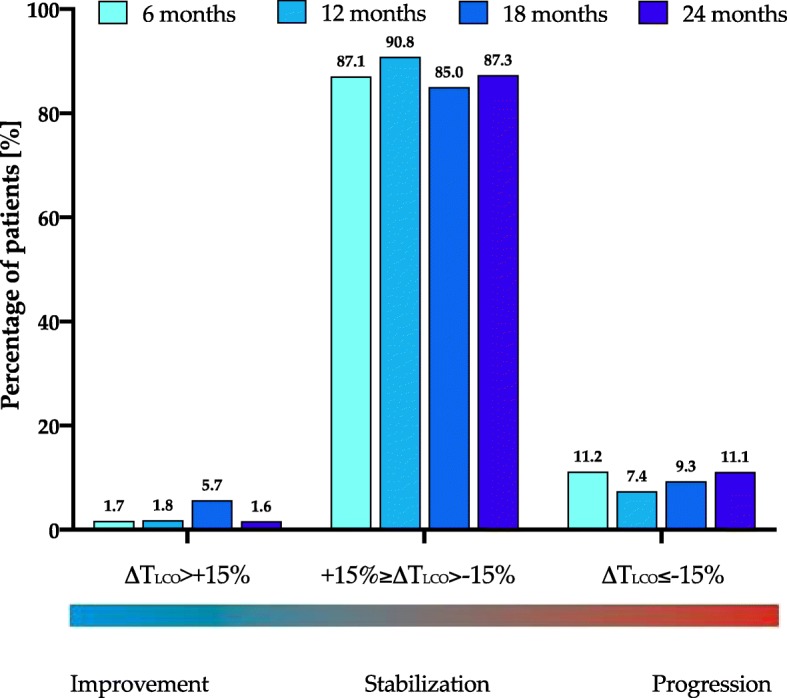
Percentage of patients with IPF experiencing significant (∆T_LCO_ >15%) improvement, stabilization (+15% ≥ ∆T_LCO_>-15%), and significant (∆T_LCO_ ≤ − 15%) decline based on the rate of changes of T_LCO_ % of predicted (∆T_LCO_) in the 6-months intervals during pirfenidone treatment

The graphical presentation of the interval subanalysis of FVC and T_LCO_ data is shown in Figs. 4 and 5.

## Discussion

In the present study, we examined retrospectively longitudinal data on clinical outcomes of pirfenidone therapy in patients with IPF in Poland under real-world conditions. The main findings of our study confirm the long-term acceptable safety and tolerability profiles of pirfenidone in the treatment of IPF and show that the large percentage of patients with IPF receiving pirfenidone experience functional stabilization and in the minority of them a functional improvement may be observed over up to 24 months of follow-up. Moreover, the Polish RWD on the pirfenidone safety and efficacy in IPF are in the line with the results of previous RCTs and other smaller retrospective and observational studies undertaken to date. To sum up, the PolExPIR study is a valuable source of evidence gathered from the large representative real-world population of patients with IPF in Poland.

Pirfenidone is an oral antifibrotic drug which has been licensed for the treatment of patients with mild-to-moderate IPF in Europe (2011) and in the United States (2014) and is currently conditionally recommended in the international clinical practice treatment guidelines [7]. Nevertheless, lack of reimbursement policy for antifibrotics in Poland resulted in very limited access to antifibrotic therapy for Polish patients with IPF, which has been clearly demonstrated by the real-life survey conducted among the Polish pulmonologists in 2016 [8].

Recently, changes in the reimbursement policy for antifibrotics in Poland have led to the wider availability of pirfenidone since January 2017 for the treatment of Polish patients with IPF [34]. The drug has become available in the frame of therapeutic program refunded by the NHF for patients with the mild-to-moderate disease based on the inclusion criteria similar to those used for the phase III pirfenidone RCT, namely the ASCEND study [5]. As a consequence of such therapeutic program eligibility criteria, the baseline demographic and clinical characteristics of patients in the PolExPIR cohort were similar to those from the ASCEND trial [5]. In both cohorts, patients had a mean age of 68 years, close to 80% of them were males, and about two-thirds were former smokers. However, slightly higher percentage of patients in the ASCEND trial had a definite UIP pattern in HRCT compared to the PolExPIR cohort (95% vs 90%, respectively), and the median baseline value of FVC % of predicted was lower in the ASCEND trial compared to the PolExPIR (68% vs 77%, respectively). Our study cohort represented real-world clinical practice population of patients with IPF with a number of coexisting comorbidities and drug therapies which could potentially exclude at least a part of them from participation in the RCTs like the ASCEND study. Lack of strict selection of patients for therapy and a more pragmatic approach is typical for real-world setting clinical practice. Therefore, the results of the PolExPIR study can be considered to be more representative of daily clinical practice than the results of RCTs.

Our analysis of drug tolerability, safety assessment, and treatment persistence confirmed well-established safety and tolerability profiles of pirfenidone [35–37]. The ADRs observed in our study were similar to those reported in pirfenidone RCTs and no new long-term use safety signals have emerged. Fatigue along with gastrointestinal and skin-related ADRs were among the most common adverse events recorded in our study. Interestingly, the most frequent ADR in the PolExPIR cohort was fatigue occurring in 35.83% of patients during the study follow-up period. Noted frequency of fatigue during pirfenidone treatment is significantly higher than the incidence of fatigue reported in RCTs, namely CAPACITY (7%) and ASCEND (21%) trials. However, even a slightly higher incidence of fatigue (38.9%) than observed in the PolExPIR cohort has been reported in the nationwide observational study undertaken in Denmark [17]. The ADRs led to permanent treatment discontinuations in 16.61% of Polish patients which, despite median longer drug exposition, is in good agreement with the rates of pirfenidone discontinuations due to ADRs reported in the CAPACITY study (15%) [4] and the ASCEND study (14.4%) [5]. Although, pirfenidone treatment discontinuation rates due to ADRs reported in the previous observational RWD studies vary significantly in the range of 1.7 to 31.8% [12, 14, 17, 19, 25–28]. The majority of discontinuations in our study related to ADRs (84%) were gastrointestinal and skin-related which is consistent with previous pirfenidone tolerance data [35–37]. No patient experienced ADRs with a fatal outcome. Treatment discontinuation rates in the PolExPIR cohort due to death (8.79%) or disease progression (6.51%) were slightly lower than in the recently published report of the French Ancillary Study (FAS) of PASSPORT registry (11.5 and 10.9% respectively) despite similar median duration of exposure to pirfenidone in both studies (17 vs 16.3 months, respectively) [26]. Surprisingly, only one patient in the PolExPIR cohort (0.33%) discontinued pirfenidone treatment due to lung transplantation. In the recently published results of an observational study of patients with IPF treated with pirfenidone in Belgium and Luxembourg (PROOF registry) the lung transplant rate for all patients during a study follow-up of 24 months was 5.6% (13 out of 233 patients) [28]. This finding confirms that improvement in cooperation between pulmonary centres and transplantation units in Poland is crucial for optimal patients outcomes in this matter.

Due to retrospective character of the PolExPIR study, it has not included any specific survival analysis. However, we did obtain valuable information on patients who died during the study follow-up. Our mortality data confirm that lung disease itself is the most common cause of death in IPF, next to cardiovascular and neoplastic disease-related deaths. These observations are in line with a recently published analysis of causes of death in patients with IPF in Finland [38].

Over 24 months of follow-up in the PolExPIR cohort PFTs values remained generally stable. Interestingly, median annual FVC decline of −20 ml in the first year and −120 ml in the second year of pirfenidone treatment observed in the PolExPIR was even less than that noted in the ASCEND trial, in which mean decline from baseline in FVC was −235 ml in the pirfenidone group over 52 weeks [5]. However, in the real-world study reporting clinical experience of the long-term use of pirfenidone in the large cohort of Japanese patients with IPF the mean decline of FVC was −30 ml and −158 ml in the first and second year of therapy, respectively [29], which is in good agreement with our study results. Moreover, a study of Tzouvelekis et al. [19] and a recent study of Vietri et al. [30] reported similar findings of worsening of FVC decline rate after 12 months of pirfenidone treatment. Taken together, ours and others data may suggest that long-term efficacy of pirfenidone in IPF may differ according to the duration of treatment. On another note, a recent observational study of Czech IPF cohort from EMPIRE registry reported a substantial stability of FVC decline rate even after 2 years of pirfenidone therapy [39]. Given the fact, that PolExPIR and other RWD studies of long-term pirfenidone use in IPF reporting worsening of FVC decline after 12 months of pirfenidone treatment are retrospective, their results should be interpreted with caution. Therefore, a well-designed, prospective, longitudinal study undertaken in the large IPF population is needed to clarify whether pirfenidone maintains its efficacy over a follow-up of more than 1 year.

The median change from baseline in % of predicted FVC was −0.68% at month 12 and −5.42% at month 24, which is definitely below the threshold of 10% decline regarded as a marker of significant disease progression. A similar observation was noted for the median change from baseline in % of predicted T_LCO_ of −4.94% at month 12 and −8.18% at month 24, which is also below clinically significant deterioration threshold of 15% decline [2]. Longitudinal evaluation of PFTs change over the study follow-up, including 6-months interval analysis of data, confirmed that the majority of patients obtained disease stabilization under pirfenidone treatment and only minority of them improved or declined in terms of PFTs results.

The decline in the 6MWT distance over the study follow-up was likewise promising. We observed no change in the 6MWT distance over the first year of treatment and the median change of only −31.5 m after 24 months from the initiation of pirfenidone therapy. However, the number of patients with available data for analysis was low. For comparison, in the phase III pirfenidone trials CAPACITY 004 and 006 the mean change in 6MWT distance based on pooled data analysis was −52.8 m in the pirfenidone group over 72 weeks [4]. Interestingly, a recently published analysis of FAS of PASSPORT registry of IPF cohort reported the mean change from baseline in 6MWT distance of 8.6 and 3.1 m at months 12 and 24, respectively [26]. It is likely, that both the PolExPIR and the FAS of PASSPORT registry results could suffer from bias related to a low number of patients with available data for longitudinal analysis of change in 6MWT distance.

However, changes in 6MWT distance after 12 and 24 months of pirfenidone therapy, observed in our cohort, are concordant with the finding of difference in FVC decline in the first and the second year of follow-up, which is supporting validity of our study results.

The main strengths of the PolExPIR study are non-sponsored, multicentre design and analysis of data collected from one of the largest real-world population of patients with IPF treated with pirfenidone to date. Very few studies undertaken worldwide provide such data from the cohorts of more than 300 patients [18, 27, 29, 39]. The median pirfenidone exposition in our study was longer than in RCTs and most of the observational studies published to date. Lack of strict inclusion and exclusion criteria characterizing RCTs allowed for a collection of data more representative for the broad population of patients with IPF typical for the real-world setting clinical practice. A broader patients population and a longer median follow-up may record a more complete picture of pirfenidone safety and effectiveness than RCTs.

The findings of our study should be considered in the context of several limitations. The most important one is the retrospective nature of the multicentre observational study. It is of note, that study participating centres were selected based on their experience in the management of patients with IPF, although the collection of clinical data and PFTs or 6MWT administration practices may have differed across those sites. This could lead to both missing data and reporting bias. Due to the study design, patients discontinuing treatment were excluded from longitudinal follow-up which may have induced bias in the reporting of survival data. The assessment of effectiveness is limited by a single-arm design and a lack of the control group. Our study efficacy data could be strengthened by pre-treatment and post-treatment comparison of the rate of decline of FVC and T_LCO_, although this was not possible due to a small number of available previous PFTs results. Despite the above limitations, in our opinion, obtained results provide confidence since safety, tolerability and efficacy data are in line with that of pirfenidone RCTs and other real-world observational studies.

## Conclusions

In conclusion, the PolExPIR study provides for the first time the real-world setting data on the longitudinal clinical outcomes of pirfenidone therapy in the large cohort of patients with IPF in Poland. Over up to 24 months of follow-up, the pulmonary function of patients with IPF receiving pirfenidone remained largely stable and ADRs led to permanent treatment discontinuations in 16.61% of patients. Taken together, the main study findings confirm pirfenidone’s long-term acceptable safety and efficacy profiles and reinforce conclusions from the previous RCTs and observational studies.

## Data Availability

The dataset analysed during the current study are available from the corresponding author on reasonable request.

## Abbreviations

6MWT: Six-minute walk test
ADRs: Adverse drug reactions
AZA: Azathioprine
CS: Corticosteroids
GAP: Gender, age, and 2 physiology variables (FVC and T_LCO_)
HRCT: High-resolution computed tomography
FEV_1_: Forced expiratory volume in 1 s
FVC: Forced vital capacity
ILDs: Interstitial lung diseases
IPF: Idiopathic pulmonary fibrosis
IQR: Interquartile range
MDT: Multidisciplinary team
NAC: N–acetylcysteine
NHF: National Health Fund
PaO_2_: Partial pressure of arterial oxygen
PFTs: Pulmonary function tests
RCTs: Randomised controlled trials
RWD: Real-world data
SD: Standard deviation
SLB: Surgical lung biopsy
SpO_2_: Percutaneous oxygen saturation
TBLB: Transbronchial lung biopsy
TBLC: Transbronchial lung cryobiopsy
T_LCO_: Transfer factor of the lung for carbon monoxide
UIP: Usual interstitial pneumonia
